# Editorial: Environmental enrichment in fish models: from mechanisms to behavior

**DOI:** 10.3389/fnbeh.2023.1177535

**Published:** 2023-05-17

**Authors:** Priscila Fernandes Silva, Fabiano Perez Menezes, Ana Carolina Luchiari

**Affiliations:** Department of Physiology and Behavior, Federal University of Rio Grande do Norte, Natal, Brazil

**Keywords:** housing conditions, anxiety, fear, animal model, welfare

Environmental enrichment (EE) refers to any modification of an animal's surroundings that enhances its adaptability and welfare in captivity. Fish species are crucial for biomedical research, particularly in the study of neuropsychiatric and neurodegenerative disorders (King, 2009; Ogi et al., 2021). However, it is important to establish standardized housing conditions and EE strategies in fish laboratories, as these can affect test procedures and experimental results. To do so, it is essential to understand the species' behavior and how laboratory housing conditions can impact it.

This Research Topic focused on the effects of EE and housing conditions on zebrafish, a popular model in biomedical science. Over 1,000 laboratories worldwide use zebrafish as a research model (zfin.org). Therefore, the zebrafish scientific community strongly urges the definition of guidelines to standardize husbandry conditions (Alestrom et al., 2019). The studies presented in this Research Topic highlight this need and suggest alternatives to EE and zebrafish housing. For instance, how social isolation affects zebrafish behavior (Onarheim et al.), the impact of fish density and tank size (Shishis et al.), the effect of infrasound on zebrafish behavior (Scatterty et al.), and how swimming in flow water alters exploratory behavior (DePasquale et al.). All of these studies used common paradigms to evaluate behavior in zebrafish, which allows comparison. The results demonstrated that different environmental factors modulate fish behavior and highlighted the necessity for further investigations on this topic. It is crucial to consider these factors when designing experiments involving zebrafish or any other fish species.

One of the reasons that explains the lack of reproducibility is the different surroundings in experimental procedures and laboratory conditions. Without standardized protocols and procedures, there may be inconsistencies in the way experiments are conducted, making it difficult to compare results across studies or to replicate findings. These inconsistencies can include differences in animal housing and handling, the types of enrichment used in different laboratories, and other factors that can affect the experimental outcomes.

Housing and enrichment are important factors to consider in fish research. Fish are often used in scientific research because they are relatively easy to maintain in laboratory conditions, have a high reproductive capacity, and can be used to study a wide range of biological processes. For example, social fish, such as zebrafish, show stress response to isolation (Shams et al., 2017), a condition many times imposed on fish due to the need for identity knowledge in experiments (Rácz et al., 2021). In these cases, physical enrichment with plants and substrate (Marcon et al., 2018), and even auditory enrichment, improves welfare and decreases anxiety-like response (Marchetto et al., 2021). Therefore, in addition to basic housing requirements, enrichment is an important consideration for fish welfare and research outcomes. Enrichment can take many forms, such as providing hiding places, changing the tank environment, or providing social interactions. Enrichment can help to reduce stress and improve the health and wellbeing of the fish, which can lead to more reliable research results.

In this Research Topic, we approach housing and enrichment in different ways that together compose important considerations in fish research. The studies presented here focused on the physical and social conditions of fish housing and their effects on fish response in the novel tank, open field, and the black/white preference test, all common paradigms to evaluate anxiety, fear, and exploratory behavior in fish. Overall, we demonstrate that different environmental factors modulate fish behavior, indicating the necessity for further investigations on such topics.

## Author contributions

PS and AL contributed substantially to the concept and made the original draft of the article. FM revised it critically for important intellectual content. All authors contributed to the article and approved the submitted version.
